# Deep learning–based diagnostic models for bone lesions: is current research ready for clinical translation?

**DOI:** 10.1007/s00330-023-10555-w

**Published:** 2024-01-08

**Authors:** Jingyu Zhong

**Affiliations:** Department of Imaging, Tongren Hospital, Shanghai Jiao Tong University School of Medicine, Shanghai, 200336 China

Unexpected bone lesions are frequently encountered on routine imaging examinations. The clinicians rely on timely and accurate diagnosis to reach an appropriate clinical decision-making. The malignant bone tumors usually need a series of treatments, while the benign ones may be safely ignored or only require follow-ups. Further, the differential diagnosis of bone tumors includes bone infections, which requires distinct management. Although report and data systems (RADS) have been developed to guide the characterization of bone lesions [1], it is still a great challenge for radiologists to make diagnosis due to their confusing appearance.

Deep learning (DL)–based models have been developed using medical images to classify bone tumors [2–6]. As one of the latest achievements in this field, Ye et al developed a multi-task DL-based model using MRI using multi-center datasets of 749 patients with primary bone tumors or infections [7]. On an external testing dataset, the model, based on T1WI and T2WI, reached an intersection over union of 0.71 and 0.65 for detection, and Dice scores of 0.75 and 0.70 for segmentation, respectively. Further, it achieved AUC of 0.959 and 0.900 for binary (bone tumor or infections) and three-category (benign, intermediate, malignant bone tumors) classifications, which surpassed junior radiologists. However, whether these models can smoothly merge into clinical workflow depends on to what extent they response to clinical needs.

Some special issues should be taken into consideration for the development of DL-based diagnosis of bone lesions. First, the primary role of imaging in clinical practice for bone lesions is not establishing an etiological diagnosis, rather answering fundamental questions regarding patient management [1]. Most of the current models aim to classify bone tumors according to their malignancy [2–6]. These models have shown increasing AUC in classification tasks. However, they neglect to take into account the confusing situation of infections. Both malignancy bone tumors and infections are classified as Bone-RADS-4, which means further treatment is necessary [1]. The model developed by Ye et al [7] has included the infections and therefore can better correspond to the Bone-RADS, and it is expected to improve the practicality and pertinence of the model.

Second, the diagnostic model that can be clinically used must have independent ability of detection, segmentation, and classification. The cropping of bone lesions using manual bounding boxes can be fast but it potentially introduces bias from the radiologists [2]. The segmentation of series of images is time-consuming, and the region of interest can be varying from one radiologist to another [6]. The detection and segmentation of bone lesions can be more difficult than other diseases such as lung nodules but not impossible [3–5, 7]. A mature DL-based model should not rely on manual inputs as the model developed by Ye et al, and the output from such a model is a truly independent second diagnosis.

Third, the current models are developed using a single imaging modality. These models showed good performance [2–7]. Nevertheless, the evaluation of bone lesions is usually conducted in a more comprehensive manner. The radiologists may ask for images of multiple modalities, take the advantages of each modality, and analyze them together to reach the final diagnosis. It is possible that the same could be done in future DL-based diagnostic models for bone lesions, integrating various imaging signs in different modalities.

The DL-based diagnostic models for bone lesions share some room for improvement, in common with other DL-based models on different clinical questions. The sufficient reporting is the first step for translation of an academic model to a practicable tool. A checklist has been published to guide the reporting of artificial intelligence in medical imaging from lesion classification to workflow optimization [8] and is also suitable for multi-task models. Ye et al [7] adopted this checklist to promote the complete reporting of the model, which allows for sufficient details to permit repetition of their work. They fulfilled all the items, except for registration. We recommended authors and reviewers of DL-based studies to adhere to this checklist. Actually, this checklist is also strongly recommended by *European Radiology* (https://www.european-radiology.org/download/5139).

Another point we would like to emphasize is the cooperation between DL-based models and human radiologists. It is common to compare the performance of models and radiologists [2–7], but it is unknown whether these models can improve the performance of radiologists, or in contrast interfere with the judgment of radiologist. It has been confirmed that the introduction of DL-based models can shorten the reading time and improve the diagnostic accuracy of inexperienced readers for coronary CT angiography [9]. It is nice to find the DL-based model surpassed junior radiologists [7]. On the other hand, it would also be a valuable model if the performance of radiologists can be improved using a concurrent reading manner in the interpretation of bone lesion cases.

The outputs of the DL-based models are reviewed and translated into reports by radiologists, but are read by clinicians, and finally, it is the patients who will be treated according to the reports. There is a need for explaining the model construction process and inference mechanism to gain the trust from the radiologists, clinicians, and patients [10]. The gradient-weighted class activation mapping method was applied by Ye et al [7] to visualize the response areas that were important for the predictions during classification. In this way, the model not only provides the answer for specific clinical question, but also explains how the answer was rendered, which is important in high-stakes decision-making fields such as medical image analysis.

In conclusion, DL-based models have the potential ability to aid the diagnosis of bone lesions, but what really matters is how to translate it into clinical practice. These models should face the special issues for bone lesions present, including treatment-aimed model development, independent diagnostic ability, and integration of multiple imaging modalities. Meanwhile, the models are also expected to be sufficiently reported, cooperate with radiologists, and finally gain trust from the stakeholders. The model built by Ye et al [7] represents one of the best current research practices in this field, but it is just at the beginning of translating academic models into practicable tools.

## Declarations

### Guarantor

The scientific guarantor of this publication is Dr. Jingyu Zhong from Department of Imaging, Tongren Hospital, Shanghai Jiao Tong University School of Medicine.

### Statistics and biometry

No complex statistical methods were necessary for this paper.

### Methodology

• commentary
